# Digital patient decision aids for endometriosis management: a scoping review protocol

**DOI:** 10.1136/bmjopen-2025-109888

**Published:** 2026-02-24

**Authors:** Océane Pittet, Marion Delvallée, Nicola Pluchino, Kevin J Selby, Glyn Elwyn, Marie-Anne Durand

**Affiliations:** 1 Department of Ambulatory Care, University of Lausanne, Unisanté, University Center for Primary Care and Public Health, Lausanne, Switzerland; 2 Research on Healthcare Performance RESHAPE, INSERM U1290, Université Claude Bernard Lyon 1, Lyon, France; 3 Pôle Santé Publique, Service Recherche et Epidémiologie Cliniques, Hospices Civils de Lyon, Lyon, France; 4 Division of Gynecology and Reproductive Medicine, Lausanne University Hospital, Lausanne, Switzerland; 5 Dartmouth College, The Dartmouth Institute for Health Policy and Clinical Practice, Lebanon, New Hampshire, USA

**Keywords:** Education, Medical, eHealth, GYNAECOLOGY, Patient Participation, PUBLIC HEALTH, Decision Making

## Abstract

**Abstract:**

**Introduction:**

Endometriosis is a chronic, oestrogen-dependent condition with a wide range of symptoms and comorbidities that significantly affect physical, emotional and psychological well-being, as well as quality of life. Women with endometriosis often face complex treatment decisions with no universally accepted gold-standard therapy. Shared decision-making, supported by patient decision aids (PtDAs), can enhance patient knowledge and promote informed preferences and decisions. Digital PtDAs, in particular, offer potential for personalised, interactive and accessible decision support. Their characteristics, development process and evaluation in endometriosis care remain underexplored. The objective of this scoping review is to map the existing literature on digital PtDAs developed for women of reproductive age (18–49) with endometriosis, across a range of healthcare and digital health contexts.

**Methods and analysis:**

This scoping review will follow the Joanna Briggs Institute (JBI) methodology for scoping reviews. A comprehensive three-step search, developed with an information specialist, will be conducted across MEDLINE (PubMed), CINAHL, Embase, Web of Science, Cochrane databases and grey literature sources. Citations will be imported into Rayyan for screening. Two independent reviewers will conduct study selection, data extraction and analysis. Data will be summarised using tables and descriptive content analysis to identify key features, development processes and evaluation methods of digital PtDAs. The review will be reported in accordance with Preferred Reporting Items for Systematic Reviews and Meta-Analyses extension for Scoping Reviews guidelines.

**Ethics and dissemination:**

This review started off in July 2025, and the anticipated end date is November 2025. We plan to disseminate this research through publications, presentations at relevant national and international conferences and meetings with relevant stakeholders. This scoping review protocol has been registered at Open Science Framework (osf.io/fp86m). As this scoping review will use data from published and publicly available sources, research ethics approval is not required.

**STRENGTHS AND LIMITATIONS OF THIS STUDY**

- Uses a comprehensive scoping review methodology guided by the JBI framework and reported in accordance with Preferred Reporting Items for Systematic Reviews and Meta-Analyses extension for Scoping Reviews standards.
- Comprehensive and inclusive search strategy across multiple databases, grey literature and decision aid inventories to capture both published and unpublished tools.
- Use of established frameworks (International Patient Decision Aid Standards criteria, Template for Intervention Description and Replication) to systematically describe patient decision aid (PtDA) characteristics, development processes and evaluation methods.
- Findings may be constrained by incomplete reporting, restricted access to full PtDAs and language limitations due to the inclusion of English-language and French-language publications only. Broad inclusion criteria across study designs and contexts may also increase heterogeneity and limit synthesis beyond descriptive mapping.
- The rapidly evolving digital health landscape may reduce the long-term applicability and transferability of the review’s findings.

## Introduction

 Endometriosis is a chronic, oestrogen-dependent and inflammatory gynaecological disease affecting approximately 10% of reproductive-age women.^1 2^ Characterised by endometrial-like tissue outside the uterus, it leads to a wide spectrum of symptoms and associated conditions. This includes pelvic pain, dysmenorrhoea and dyspareunia, often coupled with fatigue, infertility, depression and anxiety. The complex nature of endometriosis results in a significant impact on patients’ quality of life.^3^,^5^

The literature demonstrates that current diagnostic and management strategies inadequately address patients’ needs.^6 7^ Current models of care, often based on a single provider, fall short of addressing the multifaceted nature of endometriosis and need to include multidisciplinary teams to focus on treating the patients and their symptoms as a whole.^7 8^ Authors have identified a need to target psychological factors^5 9^ as well as social, emotional and sexual issues resulting from the illness^10 11^ to improve quality of life and comfort.

However, no single treatment is universally recognised as the gold standard. Each option presents unique advantages, disadvantages and varying levels of effectiveness depending on the individual patient.^12 13^ Therefore, treatment decisions in endometriosis are inherently preference-sensitive, reflecting the clinical equipoise between multiple reasonable options with no single choice clearly superior. These decisions often involve important trade-offs between benefits and risks, such as symptom relief vs side effects, surgery vs risk of recurrence, or fertility versus symptom control. The most effective approach to endometriosis treatment combines clinical characteristics with the patient’s informed preferences and needs regarding the available options.^14 15^ As a result, individual values and priorities are key in guiding treatment decisions and women with endometriosis must weigh and navigate the complexity of these options based on their personal goals, whether that be reducing symptoms like pain or achieving pregnancy, or both. To achieve this, shared medical decision-making, supported by the use of patient decision aids (PtDAs), can help patients access clear and easily understandable information about the treatment options available to them, enabling discussions with their health professionals and informed preferences and decisions.^16^ PtDAs are tools designed to support individuals in making informed healthcare decisions by presenting evidence-based information about reasonable treatment options. PtDAs may be used before, during or after a clinical consultation and can be classified into two types: pre-encounter and encounter PtDAs. PtDAs have been shown to enhance patient knowledge, reduce decisional conflict, improve accuracy in risk perception and help patients clarify their preferences.^16^ To make meaningful and personally appropriate healthcare choices, patients must be sufficiently informed and supported in evaluating what matters most to them. In the context of endometriosis—a chronic, often debilitating condition affecting women of reproductive age—PtDAs have particular relevance. Digital PtDAs such as mobile apps or web-based platforms —accessible through smartphones, laptops and other personal devices—represent a promising innovation in this space. They allow for greater interactivity, personalisation and real-time engagement, potentially improving user experience and health outcomes.^17^ Recent studies suggest that digital tools, including conversational agents or chatbots, can effectively support behaviour change and facilitate shared medical decision-making.^18 19^

Moreover, generative artificial intelligence (AI), deployed as chatbots powered by large language models (LLM), offers new opportunities to support patients by presenting complex health information in an interactive, conversational and personalised manner.^20 21^ With their ability to provide 24/7 access to guidance and educational content, these chatbots hold promise for enhancing health literacy, patient engagement and self-management. AI, including LLM, is also increasingly used by healthcare professionals, notably to develop patient instructions and educational materials^22 23^ and support shared decision-making.^24 25^

Given this development, there is interest in the development of PtDAs specifically tailored to support women affected by this condition. In particular, digital PtDAs offer potential for broader accessibility and enhanced user engagement. There is a pressing need for effective, evidence-informed digital PtDAs to support women with endometriosis in making complex treatment decisions. To develop the most optimal tool, it is essential to build on existing efforts by identifying what has already been created, how these tools were designed and evaluated, and where the current gaps lie. To better understand the current landscape of these tools, we aim to conduct a scoping review.

As this is a scoping review, we do not aim to test specific hypotheses. The overarching objective of this review is to map the existing literature on digital PtDAs developed for women of reproductive age (18–49) with endometriosis, across a range of healthcare and digital health contexts.

A preliminary search of MEDLINE, the Cochrane Database of Systematic Reviews, JBI Evidence Synthesis and PROSPERO was conducted in June 2025. We identified a related protocol entitled “Systematic Review of Patient Decision Aids for Endometriosis and Uterine Fibroids: Impact on Satisfaction, Autonomy and IPDAS Alignment”.^26^ Several key differences distinguish our review. First, their focus is not specifically on digital PtDAs, whereas our scoping review exclusively targets PtDAs delivered via digital technologies. Second, their review restricts inclusion to studies addressing predefined outcomes (satisfaction, autonomy, International Patient Decision Aid Standards (IPDAS) alignment), while our review adopts a broader scope without outcome limitations. Third, they exclude sources lacking original data, such as opinion pieces or editorials; however, we have identified three relevant digital decision aids listed in the Ottawa Inventory of Decision Aids^27^ that would be excluded under these criteria but are essential for mapping the field comprehensively. These distinctions highlight the necessity of our scoping review to thoroughly capture the landscape of digital PtDAs for endometriosis.

The aim of this scoping review is to map the existing literature on digital PtDAs developed for women of reproductive age (18–49) with endometriosis, across a range of healthcare and digital health contexts

## Methods and analysis

### Review question

The research question was developed by two researchers (OP and M-AD): What are the characteristics, development processes, and evaluation methods of PtDAs designed for women of reproductive age with endometriosis? The question focused on three main objectives:

1. To identify and describe the content and features of existing digital PtDAs for women with endometriosis, using IPDAS Minimal criteria^28^ and the Template for Intervention Description and Replication (TIDieR).^29^
2. To examine the development processes, including stakeholder involvement and use of frameworks or guidelines.
3. To summarise the evaluation methods and outcomes reported for these tools.

To ensure alignment with the research question, we defined inclusion criteria using the Population–Concept–Context framework^30^ recommended for scoping reviews.

### Inclusion criteria

#### Participants

Women with a clinical diagnosis of endometriosis of reproductive age (18–49 years old), according to the definition of the WHO.^31^

Individuals under 18 years will be excluded, as their participation would involve parents or guardians as additional decision-makers, changing the dynamics of shared decision-making compared with adult patients.^32^

#### Concept

- Studies must describe, develop, evaluate or report on PtDAs designed to support patients in making informed choices about endometriosis care. Endometriosis care encompasses pharmacological and hormonal treatments, surgical interventions, pain management strategies, fertility and reproductive options, as well as complementary or lifestyle approaches, all delivered by healthcare professionals. According to the IPDAS Collaboration,^28 33 34^ a PtDA must, at minimum: (a) clearly state the decision to be made; (b) provide evidence-based information about a health condition, the options, associated benefits, harms; (c) help patients clarify the importance they place on each option’s potential benefits and harms (implicit or explicit).
- For this review, ‘digital’ refers to any PtDA that is delivered through digital technologies and is accessible remotely (access to a digitally hosted tool—via a device—such as a phone, tablet, computer or similar). Eligible tools must be designed for use on computers, mobile devices (smartphones, tablets) or other personal digital devices that allow access outside of a single fixed location. If a tool is currently paper-based but is described in the literature as in the process of being digitised, it will be included if sufficient detail about the planned digital features is provided. The final decision on borderline cases (eg, web-based information sheets that partly meet PtDA features) will be made through discussion and consensus among the review team.
- We will also include digital PtDAs generated using conversational AI platforms to map the emerging role of generative AI in PtDA development. For this purpose, five AI platforms (ChatGPT, Gemini, Claude, Microsoft Copilot and Perplexity) will be used to generate PtDAs. These platforms have been consistently cited in industry and market analyses as widely used conversational AI tools in 2025, although relative usage estimates vary across sources.^35 36^

#### Context

Studies conducted in any healthcare setting or geographic location.

#### Types of sources

This scoping review will consider a broad range of evidence sources to comprehensively map the literature on PtDAs for women with endometriosis. Eligible sources will include both quantitative and qualitative research, as well as relevant reviews and grey literature.

Quantitative studies considered for inclusion will encompass experimental and quasi-experimental designs, such as randomised controlled trials, non-randomised controlled trials, before-and-after studies and interrupted time-series studies. Analytical observational studies, including prospective and retrospective cohort studies, case–control studies and analytical cross-sectional studies, will also be included. In addition, descriptive observational designs such as case series, individual case reports and descriptive cross-sectional studies will be considered.

Qualitative research that provides insights into the development, implementation or experiences related to digital PtDAs will be eligible. This includes studies using methodologies such as phenomenology, grounded theory, ethnography, qualitative description, action research and feminist research.

Systematic reviews that meet the inclusion criteria and contribute relevant synthesised evidence will also be included. Furthermore, grey literature and non-empirical sources—such as policy documents, websites, organisational reports and opinion papers—will be considered, particularly when they offer valuable context or information on the design, features or use of PtDAs in practice. Standalone PtDAs without accompanying research will also be included to comprehensively map the available tools.

### Exclusion criteria

#### Participants

Studies involving women who are outside the age range (under 18 or over 49 years old) or who do not have a clinical diagnosis of endometriosis will be excluded.

#### Context

Non-digital PtDAs delivered via formats that do not allow remote access through computers, mobile devices (smartphones, tablets), or other personal digital devices will be excluded. This includes tools available only in paper format or standalone desktop software without remote accessibility.

Tools that do not fulfil the minimal IPDAS definition of a PtDA will be excluded, such as interventions focused solely on lifestyle changes, social care decisions, clinical trial enrolment, or general advance directives (eg, do-not-resuscitate orders); provide only general education not linked to a specific healthcare decision; or are designed primarily to promote treatment adherence or to obtain informed consent for a recommended option rather than to support a deliberative choice among alternatives.

#### Type of sources

Studies for which neither the full text nor the PtDA can be obtained despite reasonable efforts will be excluded, as insufficient information would be available to assess eligibility or extract data.

### Methods

The proposed scoping review will be conducted in accordance with the Joanna Briggs Institute (JBI) methodology for scoping reviews.^37^ This review protocol has been registered in the Open Science Framework (OSF) registry (registration number: osf.io/fp86m).

#### Search strategy

The search strategy will aim to locate both published and unpublished studies. A three-step search strategy will be used in this review. First, an initial limited search of MEDLINE (via the PubMed interface) and CINAHL (EBSCO) was undertaken to identify articles on the topic. The text words contained in the titles and abstracts of relevant articles, and the index terms used to describe the articles were used to develop a full search strategy for: MEDLINE Ovid ALL (see online supplemental appendix 1). The search strategy was developed by the research team with the help of an information specialist. The search strategy, including all identified keywords and index terms, will be adapted for each included database and/or information source. The databases to be searched include MEDLINE (via PubMed), CINAHL Complete, Embase.com, Web of Science (Core collection), Cochrane Database of Systematic Reviews and Cochrane Central Register of Controlled Trials (CENTRAL).

To ensure comprehensive coverage, grey literature and online resources such as the online inventory of Decision Aids developed by the Ottawa Hospital Research Institute^27^ and the MAGIC app (https://www.magicevidence.org/magicapp/) will be included. The first five pages of Google search results will be screened, along with relevant websites of health organisations, government agencies and patient advocacy groups. Additionally, the first 200 results from a targeted Google Scholar search will be reviewed. The search strategy for Google Scholar will be developed and refined in collaboration with an information specialist.

To identify any additional relevant studies not captured in the database searches, we will conduct a backward citation search of the reference lists of all included full-text articles and relevant reviews. This backward citation searching will help capture additional studies that meet inclusion criteria and relevant grey literature or earlier foundational works. Any newly identified citations will be screened according to the same eligibility criteria used in previous steps.

In addition to conventional searches, we will include digital PtDAs generated using conversational AI platforms to explore the emerging role of generative AI in PtDA development. Five of the most widely used AI platforms (ChatGPT, Gemini, Claude, Microsoft Copilot and Perplexity) will be used to generate PtDAs. Each model will be provided with the standardised prompt: ‘Generate a PtDAs for individuals with endometriosis’. No additional instructions or follow-up prompts will be used beyond the initial query. PtDAs will be created either using a new account specifically for this study or, when possible, without logging into any account to limit personalised bias. This approach will allow us to capture emerging digital decision aids that may not yet be described in the scientific literature.

The search for scientific articles will be limited to English and French due to feasibility and the challenges associated with accurately translating technical content from other languages. Eligibility for digital PtDA tools will be extended to all languages as these resources can be easily accessed and translated using web browsers or translation apps. Studies published from database inception will be included to ensure a comprehensive search that captures the emergence and evolution of digitalisation, including the early development of web-based and mobile PtDA.

#### Study/source of evidence selection

Following the search, all identified citations will be collated and uploaded into Rayyan^38^ and duplicates removed. Following a pilot test, titles and abstracts will then be screened by two independent reviewers for assessment against the inclusion criteria for the review. Potentially relevant sources will be retrieved in full and their citation details imported into Rayyan. The full text of selected citations will be assessed in detail against the inclusion criteria by two independent reviewers. Reasons for the exclusion of sources of evidence at full text that do not meet the inclusion criteria will be recorded and reported in the scoping review. Any disagreements that arise between the reviewers at each stage of the selection process will be resolved through discussion or consultation with a third reviewer. The results of the search and the study inclusion process will be reported in full in the final scoping review and presented in a Preferred Reporting Items for Systematic Reviews and Meta-Analyses (PRISMA) flow diagram.^39^

For each PtDA, we will seek to obtain a copy of the tool itself along with any associated publication or documentation describing its characteristics and features. If it is not possible to access the full tool (eg, due to restricted access or it not being publicly available), data will be extracted based solely on the associated publication or report, provided that it contains sufficient detail to meet the review objectives. Standalone PtDAs without accompanying research will also be included to comprehensively map the available tools.

#### Data extraction

Data will be extracted from all sources of evidence included in the scoping review by two independent reviewers using a standardised data extraction form. The extracted data will include specific details about the participants, concept, context, study methods and key findings relevant to the review questions. We will use the data extraction form provided in the Manual for Evidence Synthesis–Scoping Reviews chapter^37^ (see online supplemental appendix 2). This form will be piloted on a small number of sources to ensure consistency and clarity. It may be modified and revised as needed during the data extraction process; any changes will be documented in the final review. Disagreements between reviewers will be resolved through discussion or consultation with a third reviewer. When required, authors of sources may be contacted to obtain missing or additional information.

#### Data analysis and presentation

In this scoping review, the extracted data will be collated, summarised and reported using tabular presentations and descriptive content analysis. Tables will display key information such as the frequency of study characteristics, features of digital PtDAs, implementation strategies and reported outcomes related to usability, accessibility, acceptability, feasibility and patient-reported experiences. We will do a descriptive content analysis to identify recurring themes, patterns and gaps within the literature.

The review will be designed, conducted and reported in accordance with the PRISMA extension for Scoping Reviews (PRISMA-ScR) guidelines^39^ to ensure transparency and methodological rigour. Furthermore, the discussion will address the implications of these findings for clinical practice, patient empowerment and future research directions aimed at optimising digital PtDA development and integration to improve health outcomes and patient experiences in endometriosis care.

#### Patient and public involvement

Two patient partners with lived experience of endometriosis will be integrated into the research team to ensure the study reflects patient perspectives and priorities. They will participate in discussions on project development and review draft manuscripts. Documents will be translated into their native language, with additional explanations of methodological concepts provided as needed. Patient partners will receive financial compensation for their contributions. Their involvement will help shape the study and ensure outputs are understandable, relevant and aligned with patient perspectives.

## Ethics and dissemination

As this scoping review will use data from published and publicly available sources, research ethics approval is not required. The review commenced in July 2025 and is expected to be completed by November 2025. Findings will be disseminated through peer-reviewed journal publications and presentations at national and international conferences focused on gynaecology, shared decision-making and digital health. Results will also be shared with key stakeholders, including clinicians, researchers and patient advocacy groups, to inform future development and evaluation of digital PtDAs for endometriosis. In addition, insights from identified research gaps will help shape future research priorities and may contribute to improving shared decision-making and clinical care for individuals with endometriosis. This protocol is registered with the OSF (osf.io/fp86m).

### Strengths and limitations of this study

The protocol of this scoping review has several strengths, including a rigorous methodology guided by the JBI framework and reporting in accordance with PRISMA-ScR guidelines. A comprehensive search strategy across multiple databases, grey literature sources and decision aid inventories will be used to identify both published and unpublished PtDAs. Established frameworks, including the IPDAS criteria and the TIDieR checklist, will be applied to systematically describe PtDA characteristics, development processes and evaluation methods. Limitations may include incomplete reporting and restricted access to full PtDAs, as well as heterogeneity arising from broad inclusion criteria, which might limit synthesis beyond descriptive mapping. Restricting the search for scientific articles to English and French may result in the omission of relevant studies, and the rapidly evolving digital health landscape may affect the long-term applicability of the findings.

## Supplementary material

10.1136/bmjopen-2025-109888 online supplemental file 1
