# Supplementary material for: Digital patient decision aids for endometriosis management: a scoping review protocol
Source: BMJ Open. 2026 Feb 24;16(2):e109888. doi: 10.1136/bmjopen-2025-109888 (PMC12933810; doi:10.1136/bmjopen-2025-109888)

# **Appendices**

### **Appendix I: Search strategy**

A comprehensive search strategy was developed and executed for MEDLINE (via Ovid). To allow for transparency, peer review, and reproducibility, the full search strategy, including all keywords and MeSH terms, Boolean operators, truncation, planned limits (e.g., language and publication date), and the number of records retrieved, is presented below:

(**Endometriosis/** OR endometrioma*.ti,ab,kf. OR endometrios*.ti,ab,kf.) **AND** (**Decision Support Techniques/** OR ((decision* **OR decid*)** ADJ**4** (aid* **OR algorithm*** OR box* **OR instrument OR intervention*** OR model* **OR process* OR material* OR method* OR program*** OR support* **OR system* OR technique* OR technolog*** OR tool*)).ti,ab,kf. **OR (interactive health communication*).ti,ab,kf. OR (interactive ADJ (internet OR online OR graphic* OR booklet*)).ti,ab,kf.** OR **(**(**Decision Making/ OR Decision Making, Shared/** **OR Patient Participation/ OR Patient Preference/ OR exp Patients/px OR Patient Satisfaction/ OR exp Health Education/ OR Educational Technology/ OR Informed Consent/ OR Choice Behavior/**OR (**decision*** OR (patient* ADJ3 (**choice*** OR engagement OR experience OR expectation* OR involvement OR participat***** OR preferenc***** OR satisfaction))).ti,ab,kf. OR **(informed consent OR risk communication OR risk assessment OR risk information).ti,ab,kf.**) **AND** (Algorithms/ OR exp Artificial Intelligence/ OR exp Internet*/ OR Internet-Based Intervention/* OR exp Medical Informatics/ *OR Information Systems/* OR exp Software/ *OR Mobile Applications/* OR ("AI" OR algorithm* OR apps OR "artificial intelligence" OR chatbot* OR "clinical decision support*" OR "computational intelligence*" OR computer* OR digital OR digitis* OR digitiz* OR ehealth OR e-health OR electronic* OR informatics OR "information system*" OR interactive OR internet OR "machine intelligence*" OR "machine learning" OR mobile OR online OR platform* OR "portable electronic app*" OR "smartphone app*" OR software* OR web).ti,ab,kf.)**)**)

The search was conducted in July 2025 and yielded 358 references.

**Appendix 2: Data Extraction Form**

Source: Appendix 10.1 — JBI data extraction instrument for source of evidence details, characteristics, and results.
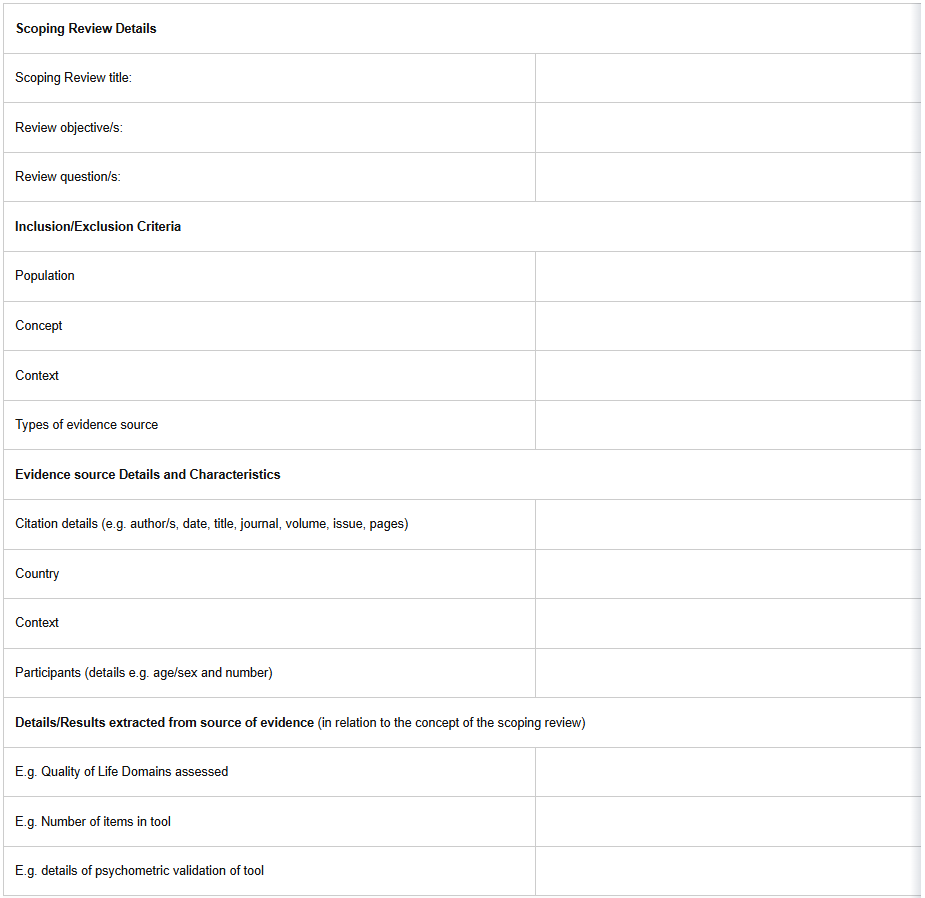

Supplement: online supplemental file 1 [file bmjopen-16-2-s001.docx]
