# Supplementary material for: Digital patient decision aids for endometriosis management: a scoping review protocol
Source: BMJ Open. 2026 Feb 24;16(2):e109888. doi: 10.1136/bmjopen-2025-109888 (PMC12933810; doi:10.1136/bmjopen-2025-109888)
Supplement: Supplementary file 2 [file bmjopen-2025-109888.reviewer_comments.pdf]

PEER REVIEW HISTORY

BMJ Open publishes all reviews undertaken for accepted manuscripts. Reviewers are asked to complete a checklist review form and are provided with free text boxes to elaborate on their assessment. These free text comments are reproduced below.

ARTICLE DETAILS

Title (Provisional)

Digital Patient Decision Aids for Endometriosis Management: A Scoping Review Protocol

Authors

Pittet, Océane; Delvallee, Marion; Pluchino, Nicola; Selby, Kevin J.; Elwyn, Glyn; Durand, Marie-Anne

---

VERSION 1 - REVIEW

Reviewer 1

Name Westermair, Anna Lisa

Affiliation University of Zurich, Institute of Biomedical Ethics and History of Medicine

Date 21-Nov-2025

COI None

Dear authors,

should the editor consider your manuscript eligible, I have only minor suggestions.

- I suggest you explain why endometriosis treatment is a preference-sensitive decision in more detail, given that this is key in justifying the relevance of PDA.

- from your introduction, it seems to me that you are focussing on stand-alone PDAs and thus would not include encounter PDAs in your review. Please clarify and justify.

- Please provide a rationale for restricting the population of interest to adults.

- In your PCC scheme, I suggest you specify what you mean by "endometriosis care", as one might subsume both treatment offered by licensed health professionals as well as scam herbal teas or something like that.

- The limitations of your design should be discussed in the main text of the protocol.

Reviewer 2

Name Donato, Helena

Affiliation **Centro Hospitalar e Universitario de Coimbra EPE, Documentation and Scientific Information Service**

Date **06-Jan-2026**

COI **None**

---

1. Restricting the literature search to French and English introduces a systematic bias. High-quality and relevant studies may exist in other languages, such as Spanish, Portuguese, or additional languages, and excluding them risks producing incomplete or skewed conclusions.

2. The Cochrane Database of Systematic Reviews should not have been searched; only CENTRAL should have been used. Searching both databases leads to duplication of results, as it mixes primary research with secondary evidence, thereby increasing the risk of double-counting and distorting the evidence base.

3. It is unclear whether the authors searched MEDLINE exclusively via the PubMed interface, or whether they searched both MEDLINE and PubMed Central (PMC). This distinction is important and should be clearly specified, as MEDLINE and PMC differ in scope and content. Clarifying the databases and interfaces used is necessary for transparency and reproducibility of the search strategy.

Apart from the points raised above, the overall methodology is sound, and both the search strategy and the search equation appear to be appropriate and well constructed.

Ideally, literature searches should be conducted across all languages. When full access to certain publications is not possible, their existence should at least be identified and transparently reported, so that readers are aware of potentially relevant evidence that was not included.

VERSION 1 - AUTHOR RESPONSE

Reviewer: 1

- **Reviewer 1, comment 1:** I suggest you explain why endometriosis treatment is a preference-sensitive decision in more detail, given that this is key in justifying the relevance of PDA.

Reviewer 1, Response 1: Thank you for this comment. We have expanded the introduction (p.4) to provide a more detailed explanation of why endometriosis treatment is a preference-sensitive decision. The additions are highlighted in bold : *"However, no single treatment is universally recognized as the gold standard. Each option presents unique advantages, disadvantages, and varying levels of effectiveness depending on the individual patient. Therefore, treatment decisions in endometriosis are inherently preference-sensitive, reflecting the clinical equipoise between multiple reasonable options with no single choice clearly*

superior. These decisions often involve important trade-offs between benefits and risks, such as symptom relief versus side effects, surgery versus risk of recurrence, or fertility versus symptom control. The most effective approach to endometriosis treatment combines clinical characteristics with the patient's informed preferences and needs regarding the available options. As a result, individual values and priorities are key in guiding treatment decisions and women with endometriosis must weigh and navigate the complexity of these options based on their personal goals, whether that be reducing symptoms like pain or achieving pregnancy, or both."

- **Reviewer 1, comment 2:** From your introduction, it seems to me that you are focussing on stand-alone PDAs and thus would not include encounter PDAs in your review. Please clarify and justify.

Reviewer 1, Response 2: Thank you for this comment. We are including pre-encounter PtDAs (PtDAs introduced to patients before clinical encounters) as well as Encounter PtDAs (Shorter, simpler PtDAs that may be used before, during, or after consultations). By standalone PDAs, we mean a PtDA tool without an associated scientific publication ("Standalone PtDAs without accompanying research ", p.7 & p.10). For more clarity, we have described in the introduction the 2 types of PtDAs: *"PtDAs may be used before, during, or after a clinical consultation, and can be classified into two types: pre-encounter and encounter PtDAs."*

- **Reviewer 1, comment 3:** Please provide a rationale for restricting the population of interest to adults.

Reviewer 1, Response 3: Thank you for this comment. We have restricted the population of interest to adults, specifically women of reproductive age (18–49 years), in line with the World Health Organization's definition (p.6). Additionally, including individuals under 18 would introduce parents or guardians as additional decision-makers, which changes the dynamics of shared decision-making. This information was added in the methods and analysis part, sub-section inclusion criteria (p.6) in order to clarify our decision : "*Individuals under 18 years were excluded, as their participation would involve parents or guardians as additional decision-makers, changing the dynamics of shared decision-making compared with adult patients."*

- **Reviewer 1, comment 4:** In your PCC scheme, I suggest you specify what you mean by "endometriosis care", as one might subsume both treatment offered by licensed health professionals as well as scam herbal teas or something like that.

Reviewer 1, Response 4: Thank you for this comment. We have clarified what is included under "endometriosis care" (p.6): *"....pharmacological and hormonal treatments, surgical interventions, pain management strategies, fertility and reproductive options, as well as complementary or lifestyle approaches, all delivered by healthcare professionals".*

- **Reviewer 1, comment 5:** The limitations of your design should be discussed in the main text of the protocol.

Reviewer 1, Response 5: We've added a "Strengths and limitations of this study" section in the main text (p.11) : *"The protocol of this scoping review has several strengths, including a rigorous methodology guided by the JBI framework and reporting in accordance with PRISMA-ScR guidelines. A comprehensive search strategy across multiple databases, grey literature sources, and decision aid inventories will be used to identify both published and unpublished patient decision aids (PtDAs). Established frameworks, including the IPDAS criteria and the TIDieR checklist, will be applied to systematically describe PtDA characteristics, development processes, and evaluation methods. Limitations may include incomplete reporting and restricted access to full PtDAs, as well as heterogeneity arising from broad inclusion criteria, which might limit synthesis beyond descriptive mapping. Restricting the search for scientific articles to English and French may result in the omission of relevant studies, and the rapidly evolving digital health landscape may affect the long-term applicability of the findings."*

Reviewer: 2

- **Reviewer 2 comment 1:** Restricting the literature search to French and English introduces a systematic bias. High-quality and relevant studies may exist in other languages, such as Spanish, Portuguese, or additional languages, and excluding them risks producing incomplete or skewed conclusions. Ideally, literature searches should be conducted across all languages. When full access to certain publications is not possible, their existence should at least be identified and transparently reported, so that readers are aware of potentially relevant evidence that was not included.

Reviewer 2, Response 1: Thank you for this comment. We have limited the search for scientific articles to English and French publications (p.9), as these are the languages our team is fluent in, and we considered that accurately translating technical content from other languages could be challenging. For digital patient decision aid (PtDA) tools, we decided to extend eligibility to all languages (e.g., Spanish, Dutch), as these online resources, such as PDFs or webpages, can be easily accessed and translated using web browsers or translation apps. These clarifications have been added to page 9 of the manuscript: "*The search for scientific articles will be limited to English and French due to feasibility and the challenges associated with accurately translating technical content from other languages. Eligibility for digital patient decision aid (PtDA) tools will be extended to all languages as these resources can be easily accessed and translated using web browsers or translation apps*." We also included this in the "strengths and limits" section (p.11); see **Reviewer 1, Response 5:** for the full paragraph.

- **Reviewer 2 comment 2:** The Cochrane Database of Systematic Reviews should not have been searched; only CENTRAL should have been used. Searching both databases leads to duplication of results, as it mixes primary research with secondary evidence, thereby increasing the risk of double-counting and distorting the evidence base

Reviewer 2, Response 2: Thank you for raising an important methodological point regarding the search strategy.

We would like to respectfully clarify and justify our decision to search the Cochrane Database of Systematic Reviews (CDSR) in addition to CENTRAL, in line with the objectives and methodological guidance for scoping reviews.

First, unlike systematic reviews, scoping reviews aim to map the breadth and nature of evidence on a topic, including both primary sources and secondary evidence. The Joanna Briggs Institute (JBI) Manual for Evidence Synthesis explicitly recommends that scoping review search strategies be as comprehensive as possible and include reviews as eligible sources of evidence. As stated in Section 10.2.5 of the JBI Manual (2024):

> *"The search strategy for a scoping review should ideally aim to be as comprehensive as possible… in order to identify both published and unpublished primary sources of evidence, as well as reviews."*

Accordingly, the inclusion of CDSR is methodologically justified within a scoping review framework.

Second, we acknowledge the reviewer's concern regarding potential duplication and double-counting. However, this risk primarily applies to quantitative synthesis in systematic reviews and meta-analyses. Our planned analysis follows established scoping review methodology, as outlined by Pollock et al. (2023), which emphasizes descriptive mapping rather than synthesis of outcomes. As such, the inclusion of systematic reviews does not distort the evidence base but rather contributes to understanding how evidence has been generated, synthesized, and used in the field.

Third, in our review, systematic reviews identified from MEDLINE and Embase are not excluded a priori. Excluding CDSR while retaining systematic reviews from other databases would therefore introduce inconsistency into the search strategy. Moreover, in the specific context of our scoping review (decision-support tools), systematic reviews can serve as an important source for identifying relevant tools, frameworks, and conceptual approaches, even when the tools themselves originate from primary studies.

Finally, all retrieved records undergo rigorous de-duplication and screening procedures, and systematic reviews are clearly identified and handled as a distinct category of evidence. This approach minimizes the risk of duplication while preserving the comprehensiveness expected of a scoping review.

For these reasons, we believe that searching CDSR in addition to CENTRAL is appropriate and methodologically sound for this scoping review.

- **Reviewer 2 comment 3:** It is unclear whether the authors searched MEDLINE exclusively via the PubMed interface, or whether they searched both MEDLINE and PubMed Central (PMC). This distinction is important and should be clearly specified, as MEDLINE and PMC differ in scope and content. Clarifying the databases and interfaces used is necessary for transparency and reproducibility of the search strategy.

Reviewer 2, Response 3: Thank you for this comment. We have clarified the databases and interfaces used in the search strategy. Specifically, we now specify that an initial limited search of MEDLINE (via the PubMed interface) and CINAHL (EBSCO) was undertaken to identify articles on the topic. We also clarified that the text words contained in the titles and abstracts of relevant articles, and the index terms used to describe the articles were used to develop a full search strategy for: MEDLINE Ovid ALL *(see Appendix 1*). This distinction has been explicitly added to the manuscript (p.8).

Other:

Following recommendations from our Steering Committee, we have added the use of generative AI platforms in the protocol. In recent years, the use of AI-powered conversational agents has become increasingly widespread in the general population, offering new ways to access health information and understand medical conditions, and creating novel opportunities to support patient empowerment.

We've added the following paragraphs in the introduction (p.5): *"Moreover, generative artificial intelligence (AI), deployed as chatbots powered by large language models, offers new opportunities to support patients by presenting complex health information in an interactive, conversational, and personalized manner. With their ability to provide 24/7 access to guidance and educational content, these chatbots hold promise for enhancing health literacy, patient engagement, and self-management. Large language models are also increasingly used by healthcare professionals, notably to develop patient instructions and educational materials."* as well as in the Methods and analysis section (p.7):

"We will also include digital PtDAs generated using conversational Artificial Intelligence (AI) platforms to map the emerging role of generative AI in PtDA development. For this purpose, five AI platforms, ChatGPT, Gemini, Claude, Microsoft Copilot, and Perplexity, will be used to generate PtDAs. These platforms have been consistently cited in industry and market analyses as widely used conversational AI tools in 2025, although relative usage estimates vary across sources." and p.9:

"In addition to conventional searches, we will include digital PtDAs generated using conversational Artificial Intelligence (AI) platforms to explore the emerging role of generative AI in PtDA development. Five of the most widely used AI platforms (ChatGPT, Gemini, Claude,

Microsoft Copilot, and Perplexity) will be used to generate PtDAs. Each model will be provided with the standardized prompt: "Generate a patient decision aid for individuals with endometriosis." No additional instructions or follow-up prompts will be used beyond the initial query. PtDAs will be created either using a new account specifically for this study or, when possible, without logging into any account to limit personalization bias. This approach will allow us to capture emerging digital decision aids that may not yet be described in the scientific literature."
